# In Vitro Bioactivity and Antibacterial Effects of a Silver-Containing Mesoporous Bioactive Glass Film on the Surface of Titanium Implants

**DOI:** 10.3390/ijms23169291

**Published:** 2022-08-18

**Authors:** Yueh-Ching Wang, Sheng-Hui Lin, Chi-Sheng Chien, Jung-Chang Kung, Chi-Jen Shih

**Affiliations:** 1 Department of Fragrance and Cosmetic Science, College of Pharmacy, Kaohsiung Medical University, Kaohsiung 80708, Taiwan; 2 Department of Orthopedics, Chi Mei Medical Center, Tainan 71004, Taiwan; 3 Department of Leisure and Sports Management, Far East University, Tainan 74448, Taiwan; 4 Department of Electrical Engineering, Southern Taiwan University of Science and Technology, Tainan 71073, Taiwan; 5 Drug Development and Value Creation Research Center, Kaohsiung Medical University, Kaohsiung 80708, Taiwan; 6 School of Dentistry, College of Dental Medicine, Kaohsiung Medical University, Kaohsiung 80708, Taiwan; 7 Department of Dentistry, Kaohsiung Municipal Ta-Tung Hospital, Kaohsiung 80145, Taiwan; 8 Department of Medical Research, Kaohsiung Medical University Hospital, Kaohsiung 80708, Taiwan

**Keywords:** peri-implantitis, sol-gel technique, spin coating method, silver-containing bioactive glass, titanium implant, antibacterial film

## Abstract

Peri-implantitis is defined as a bacterial infection-induced inflammation and suppuration of soft and hard tissues surrounding a dental implant. If bacteria further invade the alveolar bone, they can easily cause bone loss and even lead to the early failure of a dental implant surgery. In the present study, an 80SiO_2_–15CaO–5P_2_O_5_ mesoporous bioactive glass film system containing 1, 5, and 10 mol% of silver was prepared on titanium implant discs (MBG–Ag–coated Ti) using sol-gel and spin coating methods. The wettability and adhesion strength of the films were evaluated using contact angle measurements and adhesion strength tests, respectively. The phase composition, chemical bonding, morphology, and oxidation states of the films were analyzed via X-ray diffraction (XRD), Fourier transform infrared spectroscopy (FTIR), scanning electron microscopy (SEM), energy-dispersive X-ray spectroscopy (EDS), and X-ray photoelectron spectroscopy (XPS). In vitro bioactivity analysis of the films was performed by immersion in a simulated body fluid (SBF) for 24 h. Disk diffusion tests were performed on the early colonizing bacteria *Aggregatibacter actinomycetemcomitans* and *Streptococcus mutans* to evaluate the antibacterial ability of the films. A silver-containing mesoporous bioactive glass film with excellent biocompatibility and antibacterial properties was successfully prepared.

## 1. Introduction

Dental implants have become a standard and predictable therapy for providing adequate support for the replacement of missing teeth. A success rate of 76–84% in patients with risk factors was reported in a study by F. De Angelis [1]. Nevertheless, increasing evidence of early implant surgical failure and late peri-implantitis has been reported, representing two major frequent complications that can lead to implant loss [2].

Peri-implantitis is a pathological symptom that develops from peri-implant mucositis. In the early stages, bacteria invade and adhere to the surface of the implant from the junction of the crown and soft tissue. A biofilm formation normally involves three steps, the first of which requires the adhesion and attachment of the initial colonizers to the surface. The initial colonizing bacteria are streptococci and actinomycetes. The continuous proliferation of bacteria forms a biofilm. After the biofilm matures, the bacteria begin to secrete endotoxins, which trigger an inflammatory response in the surrounding tissue of the implant. At this stage, it is reversible peri-implant mucositis. If the bacteria continue to infect, the alveolar bone is lost. This is termed as peri-implantitis [3]. The prevalence of peri-implantitis depends on risk factors such as a lack of good oral hygiene, long-term smoking, periodontal disease, or diabetes. In patients with risk factors, the prevalence rate is as high as 53.5%, with the recurrence rate being extremely high [4]. Therefore, it is important to control the growth and attachment of the initial colonizer on the implant surface, thereby disrupting the subsequent pathogenic bacterial attachment and preventing bacterial biofilm formation on the implant surface.

Brånemark proposed intraosseous implantation to be the functional and structural dependence of bone and load-bearing implants, a concept later called “osseointegration” [5]. Consequently, titanium and its alloys were widely used as implants in the dental field [6]. The commercial pure titanium used in dental implants is an α-phase titanium metal. Commercial pure titanium approved by ASTM is divided into four grades: grade I, grade II, grade III, and grade IV, according to the content of trace elements. Grade IV is the titanium having the best elastic modulus and tensile strength and is widely used for dental implants. Dental implants often undergo surface modification to improve surface roughness and accelerate osseointegration.

Sandblasted/large-grit/acid-etched (SLA) treatment has been widely applied to modify surfaces for dental titanium implants. According to the literature, SLA surfaces are hydrophobic, resulting in diminished cell adhesion [7]. Therefore, next-generation Ti-based implant coatings should exhibit multifunctionality such as enhanced hydrophilicity and antibacterial and osteogenic activity.

Xiaotong Ye et al. used mesoporous bioactive glass (MBG) composed of 80Si–15Ca–5P as the film material and prepared films by spin coating on Ti6Al4V scaffolds to improve their biological activity [8]. The MBG film scaffolds were immersed in a simulated body fluid (SBF) for 7 days, and the subsequent results showed well-produced hydroxyapatite, confirming that the addition of MBG film helped to increase its bioaffinity. Samuel F. Robertson et al. used the sol–gel technique to coat the hydroxyapatite composite strontium material (Sr/HA) on a Ti-6Al-4V substrate and provided a layer of titanium dioxide nanotubes as an intermediate layer to improve the adhesion of the film layer [9]. The Sr/HA film exhibited good cell viability in the MTT assay.

In related research, antibiotics have been used as an antibacterial source for implants, e.g., Wongsuwan et al. used liposomes to coat minocycline, which was then coated on the surface of a titanium substrate using a sprayer [10]. F. Jahanmard et al. loaded rifampicin and vancomycin into polylactic-co-glycolic acid (PLGA) nanofibers for electrospinning and coating titanium implants [11]. Although antibiotics possess excellent antibacterial abilities, the abuse or misuse of antibiotics can easily lead to the emergence of drug-resistant bacteria. Antibacterial metal ions have been used as coatings, among which nano-silver particles exhibit the best antibacterial effect. Physical vapor deposition [12] or chemical chelation methods [13] can impart antibacterial properties to the surface of a titanium substrate but cannot provide a slow-release effect or improve plant biocompatibility.

MBGs have been developed as a new class of bioactive materials and present an ordered mesoporous channel structure, which exhibits superior bioactivity compared to conventional bioactive glasses (BGs). In our recent study, silver-containing MBG was successfully synthesized to confer antibacterial activity to these bioactive materials.

The aim of this study was to apply a silver-containing MBG coating by spin coating on the surfaces of SLA Ti alloy scaffolds to improve their surface bioactivity and antibacterial activity. The surface morphology and chemical composition of the coating and their effects on the bioactivity and antibacterial activity of the coating were investigated. In addition, the wettability and adhesion strength of the MBG–coated SLA titanium were evaluated.

## 2. Results

### 2.1. Properties of Silver-Containing Mesoporous Bioactive Glass Film

#### 2.1.1. Wettability Analysis of Silver-Containing Mesoporous Bioactive Glass Film

**Figure 1:**
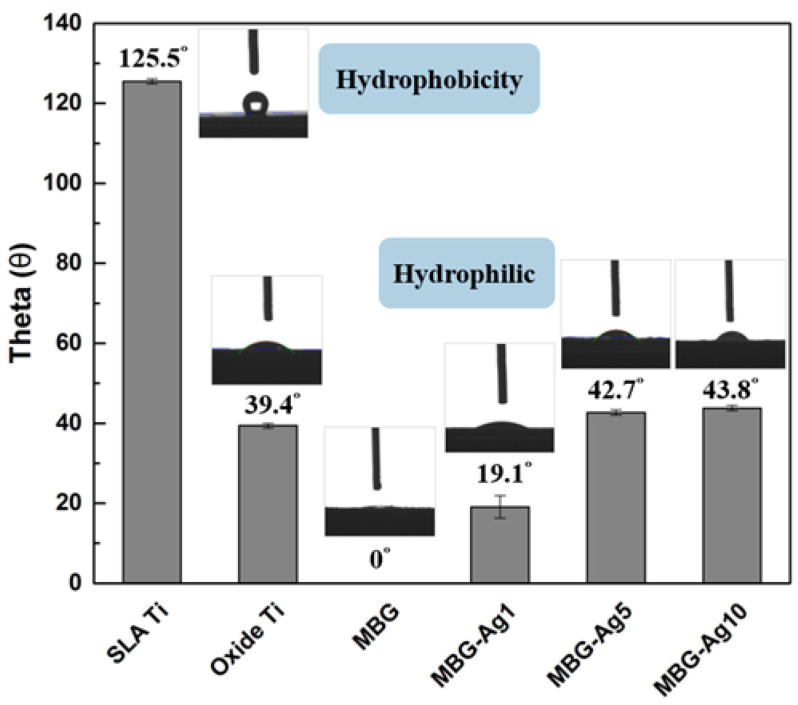
Contact angle measurement of coated specimens (N = 20) (*p* < 0.05).

In this study, a contact angle measurement system was used to analyze the surface wettability of Ag-containing mesoporous bioactive glass films. Distilled water was used as the test liquid. Referring to the literature by Giridhar et al., the hydrophilicity and hydrophobicity were evaluated according to different wetting angles [14]. The average value and standard deviation were calculated for 20 replicates, as shown in Figure 1.

From Figure 1, it can be seen that the contact angle of the control group SLA Ti specimen is 125.5°, which is an incomplete wetting surface. According to Wenzel’s theory [15], when the surface roughness is higher, the droplet is more hindered when sliding; therefore, it is easy to form an incomplete wetting surface. Since the titanium oxide layer on the surface was hydrophilic, the contact angle of the oxide Ti specimen reduced to 39.4°, resulting in a well-wetted surface. The contact angle measurement results of MBG–coated Ti and MBG–Ag1–coated Ti are 0° and 19.1°, respectively, indicating spreading and a good wetting surface. The contact angles of MBG–Ag5–coated Ti and MBG–Ag10–coated Ti were 42.7° and 43.8°, respectively. Because the hydrophilicity of bioactive glass is greater than that of nano-silver particles, when the number of nano-silver particles on the surface of MBG–Ag–coated Ti increased, the contact angle also slightly increased, causing the superhydrophilic surface to form a hydrophilic surface.

#### 2.1.2. Adhesion Strength Analysis of Silver-Containing Mesoporous Bioactive Glass Film

The film was graded according to the ASTM D–3359 standard based on the amount of film removed by the tape. The Burger’s test scale ranges from 0 (the film outside the pattern has a peeling phenomenon) to 5 (the film has no peeling or sticking phenomena). When using method A, one should add an “A” after the level such as 3A. Similarly, when using method B, one should add a “B” after the level such as 3B.

**Figure 2:**
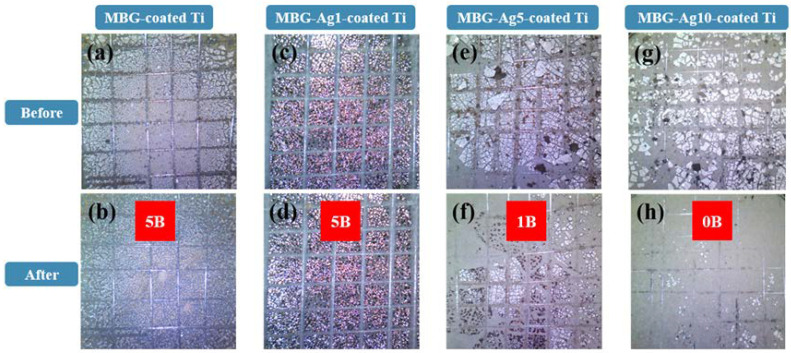
Adhesion strength analysis by Burger’s test (ASTM D–3359) of MBG–coated Ti (**a**) before, (**b**) after test; MBG–Ag1–coated Ti (**c**) before, (**d**) after test; MBG–Ag5–coated Ti (**e**) before, (**f**) after test; and MBG–Ag10–coated Ti (**g**) before, (**h**) after test (N = 3).

The Burger’s test results in Figure 2a show that before the test of MBG–coated Ti, the edge of the film was lumpy and there were signs of fall-off. After the test, as shown in Figure 2b, relatively finer crystals were observed, and the Ti substrate was not exposed. The degree of film peeling was 0%, corresponding to the 5B level. Observing the condition of MBG–Ag1–coated Ti after the test in Figure 2d, the degree of film peeling on the titanium implant specimen was 0%, corresponding to the 5B level. In Figure 2e, before the test, the MBG–Ag5–coated Ti surface exhibited evident film peeling. In Figure 2f, the degree of film peeling after the test was 35–65%, corresponding to the 1B level. In Figure 2g, before the test, the MBG–Ag10–coated Ti surface exhibited evident film peeling. In Figure 2h, the degree of film peeling after the test was greater than 65%, corresponding to the 0B level.

### 2.2. Surface Characterization Analysis of Silver-Containing Mesoporous Bioactive Glass Film

#### 2.2.1. XRD Spectra Analysis of Silver-Containing Mesoporous Bioactive Glass Film

**Figure 3:**
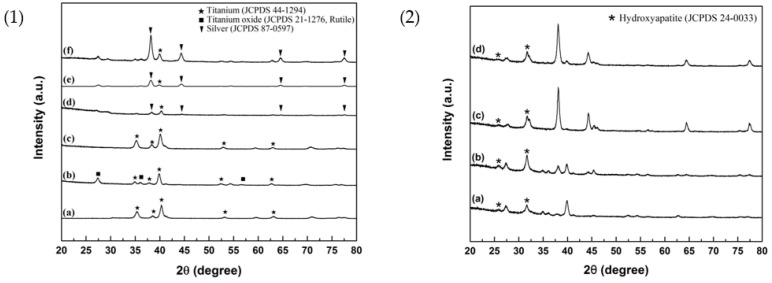
(**1**) XRD patterns of (a) SLA Ti, (b) Oxide Ti, (c) MBG–coated Ti, (d) MBG–Ag1–coated Ti, (e) MBG–Ag5–coated Ti, and (f) MBG–Ag10–coated Ti. (**2**) XRD patterns of (a) MBG–coated Ti, (b) MBG–Ag1–coated Ti, (c) MBG–Ag5–coated Ti, and (d) MBG–Ag10–coated Ti after immersion in the SBF solution for 24 h.

The XRD patterns of silver-containing mesoporous bioactive glass film are shown in Figure 3(1). Figure 3(1-a) shows the XRD pattern of SLA Ti specimen (control group). Figure 3(1-b) shows the XRD pattern of oxide Ti. Figure 3(1-c) shows the XRD pattern of MBG–coated Ti. Figure 3(1-d) shows the XRD pattern of MBG–Ag1–coated Ti containing 1 mol% Ag. Figure 3(1-e) shows the XRD pattern of MBG–Ag5–coated Ti containing 5 mol% Ag. Figure 3(1-f) shows the XRD pattern of MBG–Ag10–coated Ti containing 10 mol% Ag.

In Figure 3(1-a), the XRD pattern of the SLA Ti specimen (control group) shows diffraction peaks at 35.1°, 38.4°, 40.1°, 53°, and 62.9°. Upon comparison with JCPD 44–1294 corresponding to titanium, these diffraction peaks were assigned to the (100), (002), (101), (102), and (110) crystallographic planes, respectively. In the Figure 3(1-b), the XRD pattern of oxide Ti appears at 27.4°, 36.1°, and 56.6°, in addition to the diffraction peaks corresponding to titanium. Upon comparison with JCPD 21–1276 corresponding to the rutile phase of titanium oxide, these diffraction peaks were assigned to the (110), (101), and (220) crystallographic planes, respectively. Observing the XRD analysis results of MBG–coated Ti in Figure 3(1-c), its diffraction peaks are similar to those of the SLA Ti control group, and there are no calcium silicate crystal peaks, confirming that MBG–coated Ti has a glass structure instead of a ceramic one. Figure 3(1-d) shows the XRD patterns of MBG–Ag1–coated Ti containing 1 mol% Ag. Its diffraction peaks were observed at 38.1°, 44.3°, 64.4°, and 77.4°. Upon comparison with JCPD 87–0597 corresponding to silver, these diffraction peaks were assigned to the (111), (200), (220), and (311) crystallographic planes, respectively. With the increase in silver composition, the XRD results of MBG–Ag5–coated Ti in Figure 3(1-e) and MBG–Ag10–coated Ti in Figure 3(1-f) exhibit a higher crystalline strength compared with that in Figure 3(1-d). This confirmed that the silver-containing mesoporous bioactive glass film prepared in this study had a glass structure containing silver crystals. As the Ag content increased, the crystalline strength also increased.

#### 2.2.2. FTIR Spectra Analysis of Silver-Containing Mesoporous Bioactive Glass Film

**Figure 4:**
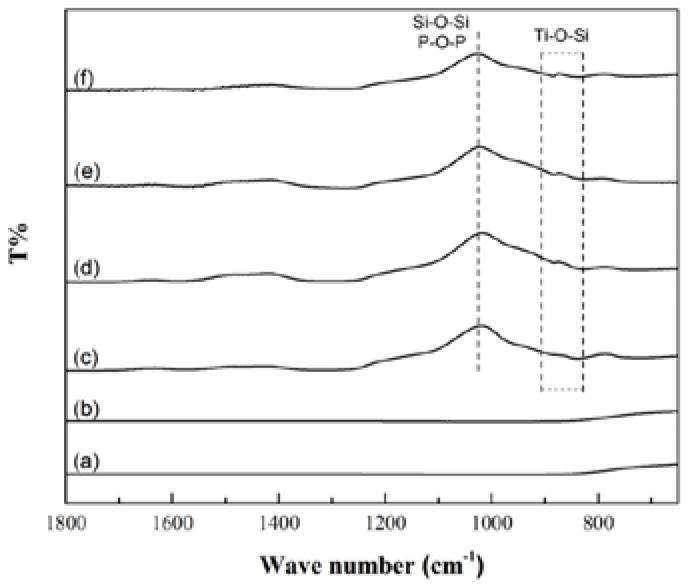
FTIR patterns of (a) SLA Ti, (b) Oxide Ti, (c) MBG–coated Ti, (d) MBG–Ag1–coated Ti, (e) MBG–Ag5–coated Ti, and (f) MBG–Ag10–coated Ti.

In this study, FTIR was used to measure the bonding of the films. From the FTIR analysis, results are shown in Figure 4.

#### 2.2.3. SEM and EDX Analysis of Silver-Containing Mesoporous Bioactive Glass Film

**Figure 5:**
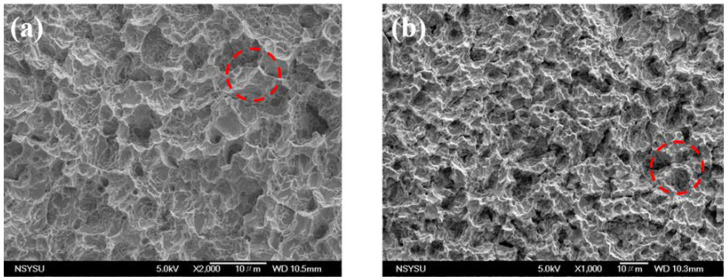
SEM images of control groups (**a**) SLA Ti, and (**b**) Oxide Ti.

Figure 5 shows the SEM images of the control groups. Figure 5a shows the surface of the SLA titanium implant, wherein there are many holes of different sizes with sharp edges (circles in the picture). Figure 5b shows the surface of SLA Ti after the oxidation treatment, wherein the edges of the holes are smoother due to passivation (circled in the figure).

**Figure 6:**
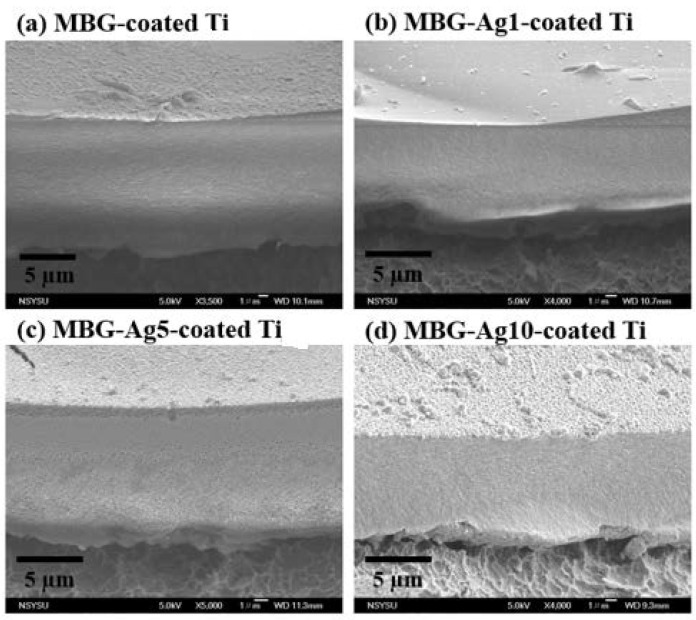
SEM images of (**a**) MBG–coated Ti, (**b**) MBG–Ag1–coated Ti, (**c**) MBG–Ag5–coated Ti, and (**d**) MBG–Ag10–coated Ti (N = 15).

The SEM images in Figure 6 show the cross-section morphology of each specimen. After measuring SEM images, it is found that the film thickness of MBG–Ag–coated Ti is between 6–18 µm. It is also observed that there were no cracks in the cross-section of the films, showing a uniform and dense pattern, and the nano-silver particles on the surface became denser with the addition of silver composition.

**Figure 7:**
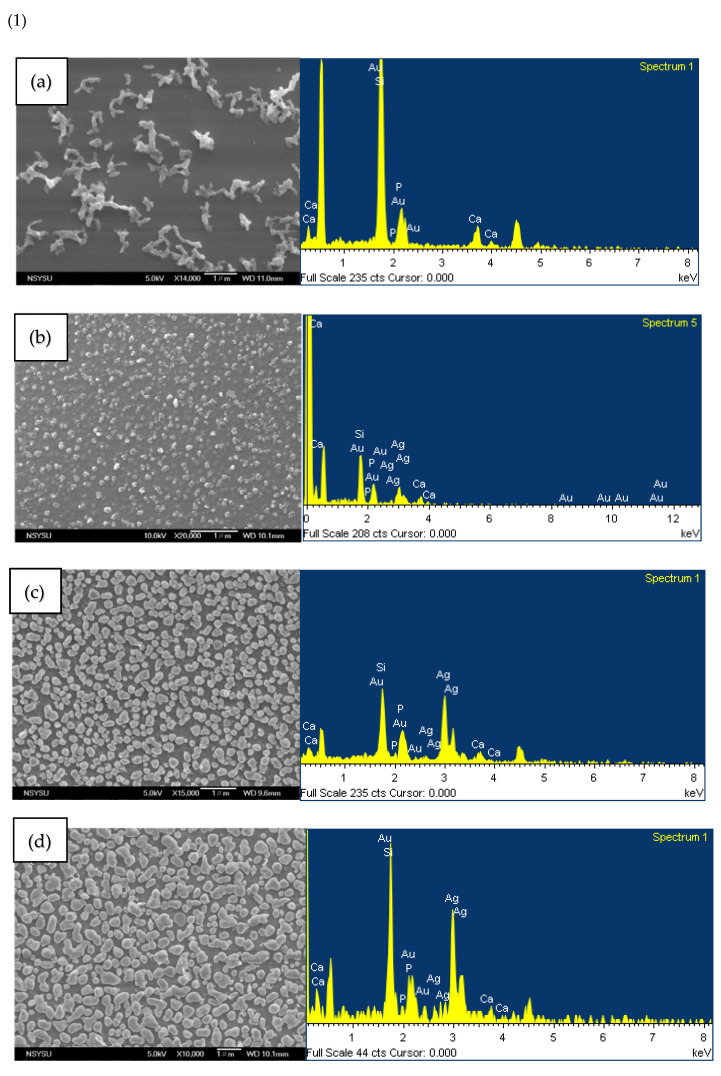

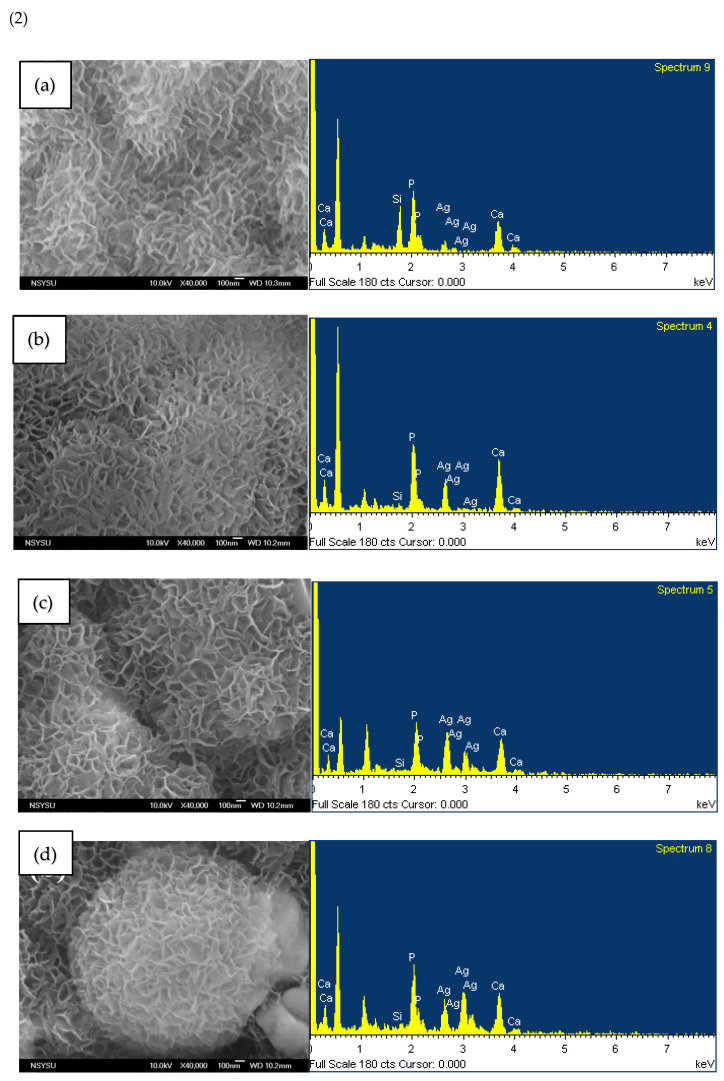
(**1**) SEM images and EDS results of (a) MBG–coated Ti, (b) MBG–Ag1–coated Ti, (c) MBG–Ag5–coated Ti, and (d) MBG–Ag10–coated Ti. (**2**) SEM images and EDS results of (a) MBG–coated Ti, (b) MBG–Ag1–coated Ti, (c) MBG–Ag5–coated Ti, and (d) MBG–Ag10–coated Ti after immersion in SBF for 24 h.

The SEM images in Figure 7(1) show the deposits on the surface of each group. As shown in Figure 7(1-a), the MBG-coated Ti surface comprises irregular deposits, presumed to be those of silicates. Figure 7(1-b)–(1-d) show that with the increase in silver composition, the deposits are spherical particles and have a gradually increasing trend. The particle sizes of MBG–Ag1–coated Ti, MBG–Ag5–coated Ti, and MBG–Ag10–coated Ti were found to be ~0.14 ± 0.03, 0.27 ± 0.03, and 0.53 ± 0.09 μm, respectively.

Each group in Figure 7(1) contained silicon, calcium, and phosphorus. The EDS results in Figure 7(1-a) show that the MBG–coated Ti has silicon, calcium, and phosphorus atomic number ratio of 74.11, 15.58, and 0.83 at%, respectively. The matrix of MBG–coated Ti is observed to be silicon. In addition, the atomic number ratio of Au is 9.48 at%, and gold atoms were obtained during the gold coating process. After adding 1 mol% of Ag, the EDS results in Figure 7(1-b) show that the MBG-Ag1-coated Ti has silicon, calcium, phosphorus, and silver atomic number ratios of 50.08, 13.07, 2.21, and 20.73 at%, respectively. After adding 5 mol% of Ag, the EDS results in Figure 7(1-c) show that the MBG–Ag5–coated Ti has silicon, calcium, phosphorus, and silver atomic number ratios of 35.96, 10.29, 0.89, and 42.76 at%, respectively. After adding 10 mol% of Ag, the EDS results in Figure 7(1-d) show that the MBG–Ag10–coated Ti has silicon, calcium, phosphorus, and silver atomic number ratios of 39.82, 7.67, 0.54, and 43.70 at%, respectively.

#### 2.2.4. XPS Analysis of Silver-Containing Mesoporous Bioactive Glass Film

In this study, XPS was used to evaluate the effect of MBG–Ag–coated Ti with different Ag compositions on the Ag^+^/Ag^0^ ratio. After a full-spectrum scan by XPS, the Ag 3d region was scanned in a narrow region to obtain the range from 365 to 380 eV, and the positions of Ag(1) and Ag(0) were obtained after peak separation using the CasaXPS software.

**Figure 8:**
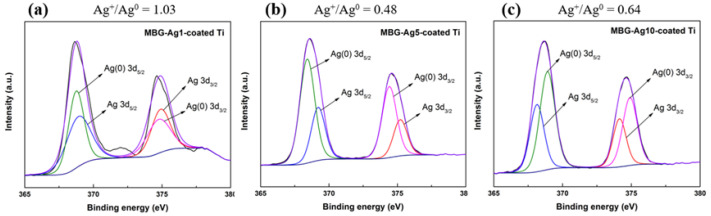
XPS patterns of (**a**) MBG–Ag1–coated Ti, (**b**) MBG–Ag5–coated Ti, and (**c**) MBG–Ag10–coated Ti.

From Figure 8a and Table 1, it can be seen that Ag(1) on the surface of MBG–Ag1–coated Ti is located at 374.90 eV and 368.90 eV with a relative ratio of 50.82% and Ag(0) is located at 374.75 eV and 368.75 eV with a relative ratio of 49.18%. Subsequently, the composition ratios of Ag(1) and Ag(0) on the surface of MBG–Ag1–coated Ti are observed to be approximately the same. From Figure 8b and Table 1, it can be seen that Ag(1) on the surface of MBG–Ag5–coated Ti is located at 375.21 eV and 369.21 eV with a relative ratio of 32.3%, and Ag(0) is located at 374.41 eV and 368.41 eV with a relative ratio of 67.7%. It is observed that the proportion of Ag(0) increases gradually, presumably due to the oxidation reaction of the film with oxygen in the air after heat treatment, such that Ag^+^ loses electrons to form Ag^0^. From Figure 8c and Table 1, it can be seen that Ag(1) on the surface of MBG-Ag10-coated Ti is located at 374.14 eV and 368.14 eV with a relative ratio of 38.85% and Ag(0) is located at 374.90 eV and 368.90 eV with a relative ratio of 61.15%. From the above results, it can be seen that when the Ag composition in MBG–Ag–coated Ti was 1, 5, and 10 mol%, the Ag^+^/Ag^0^ ratios were 1.03, 0.48, and 0.64, respectively. The Ag^+^/Ag^0^ ratio was observed to decrease with increasing silver content. Furthermore, compared with that of the MBG–Ag powder, the Ag^+^/Ag^0^ ratios of MBG–Ag1, MBG–Ag5, and MBG–Ag10 powders were 0.44, 0.19, and 0.11, respectively [16]. It can thus be seen that under the same volume, both the film and powder exist in the form of a high percentage of Ag_2_O oxidation state. Owing to the higher surface area of the film than that of the powder, the degree of oxidation increased.

### 2.3. In Vitro Bioactivity Assay Analysis of Silver-Containing Mesoporous Bioactive Glass Film

In this study, XRD was used to identify the crystalline compositions of the surfaces of MBG–Ag–coated Ti, observe whether MBG–Ag–coated Ti had the ability to induce the formation of hydroxyapatite after immersion in SBF solution for 24 h, and evaluate the in vitro biological activity of MBG–Ag–coated Ti with different silver compositions. The XRD results are shown in Figure 3(2).

Figure 7(2-a) shows the SEM image of the surface morphology of the MBG-coated Ti film after immersion in SBF for 24 h, indicating layered stacked HAp crystals on the surface. Comparing the EDS results of Figure 7(2-a) with those of Figure 7(1-a), it was observed that the silicon atomic number ratio decreased from 74.11 to 21.81 at%, because the silicon matrix was covered with HAp crystals. The calcium and phosphorus atomic number ratios increased from 15.58 to 41.33 at% and 0.83 to 37.07 at%, respectively, indicating that MBG-coated Ti effectively induced the formation of both calcium and phosphorus ions. Figure 7(2-b) shows the SEM image of the surface morphology of the MBG–Ag1–coated Ti film after immersion in SBF for 24 h, indicating that the surface has layered stacked HAp crystals similar to those on the MBG–coated Ti surface. Comparing the EDS results of Figure 7(2-b) with those in Figure 7(1-b), it was observed that the silicon atomic number ratio decreased from 50.08 to 2.68 at%, calcium atomic number ratio increased from 13.07 to 56.6 at%, and phosphorus atomic number ratio increased from 2.21 to 40 at%. Figure 7(2-c) shows the SEM image of the surface morphology of the MBG–Ag5–coated Ti film after immersion in SBF for 24 h, indicating that the surface has layered stacked HAp crystals similar to those in the first two groups. Comparing the EDS results of Figure 7(2-c) with those of Figure 7(1-c), it was observed that silicon could not be detected owing to excess HAp covering the matrix. The calcium and phosphorus atomic number ratios increased from 35.96 to 49.77 at% and 0.89 to 39.18 at%, respectively. Figure 7(2-d) show the SEM image of the surface morphology of the MBG–Ag10–coated Ti film after immersion in SBF for 24 h, indicating that the surface has layered stacked HAp crystals similar those in the first three groups, all of which are crystalline-like spherical clusters stacked layer by layer.

### 2.4. Antibacterial Activity Analysis of Silver-Containing Mesoporous Bioactive Glass Film

In this study, the disk diffusion test was used to evaluate the antibacterial ability of MBG–Ag–coated Ti with different Ag compositions. The diameter of the titanium disk was 15 mm and bacterial concentration of 10^7^ CFU/mL was used as the test condition.

#### 2.4.1. Disk Diffusion Test Analysis against *Aggregatibacter actinomycetemcomitans*

**Figure 9:**
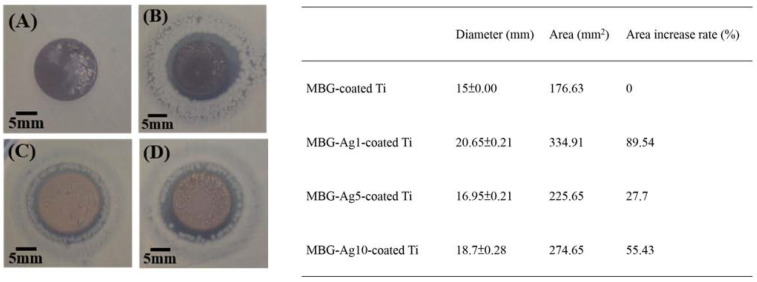
The inhibition zones of (**A**) MBG–coated Ti, (**B**) MBG–Ag1-coated Ti, (**C**) MBG–Ag5–coated Ti, and (**D**) MBG–Ag10–coated Ti against 10^7^ CFU/mL *Aggregatibacter actinomycetemcomitans*.

As shown in Figure 9A, the result of the disk diffusion test for the control group MBG–coated Ti exhibits no inhibition zone around itself and indicates that the normal growth morphology of *A. a.* with bacterial concentration of 10^7^ CFU/mL was yellow and dense, with a single colony appearing radially. In Figure 9B, a special type of inhibition zone is observed around MBG–Ag1–coated Ti, i.e., in addition to the clean and sterile zone around the specimen, there is a sparse colony around the periphery. It is a zone with sub-inhibitory concentrations of Ag. In this zone, the concentration of silver is not enough to kill bacteria, but enough to retain their growth. In the disk diffusion test results of Figure 9C (MBG–Ag5–coated Ti) and Figure 9D (MBG–Ag10–coated Ti), peripheral sparse colonies also occur. These specimens coated silver-containing mesoporous bioactive glass films with a silver composition of 1 mol%, 5 mol%, and 10 mol% against *Aggregatibacter actinomycetemcomitans* with bacterial concentration of 10^7^ CFU/mL do not reach a minimum bactericidal concentration (MBC).

After measurement and calculation, the diameter of the inhibition zone of MBG–coated Ti in Figure 9A was determined to be 15 mm and its area was 176.63 mm^2^, equivalent to the diameter and area of the titanium disk. Measured and calculated from Figure 9B,D, the inhibition zone diameters of MBG–Ag1–coated Ti, MBG–Ag5–coated Ti, and MBG–Ag10–coated Ti were 20.65 ± 0.21, 16.95 ± 0.21, and 18.7 ± 0.28 mm, respectively, while their inhibition zone areas were 334.91, 225.65, and 274.65 mm^2^, respectively. Among them, the increment rate of the antibacterial zone area with a silver composition of 1 mol% was 89.54%, but as the silver content increased to 5 and 10 mol%, the increment rate of the antibacterial zone area decreased to 27.7% and 55.43%, respectively.

#### 2.4.2. Disk Diffusion Test Analysis against *Streptococcus mutans*

**Figure 10:**
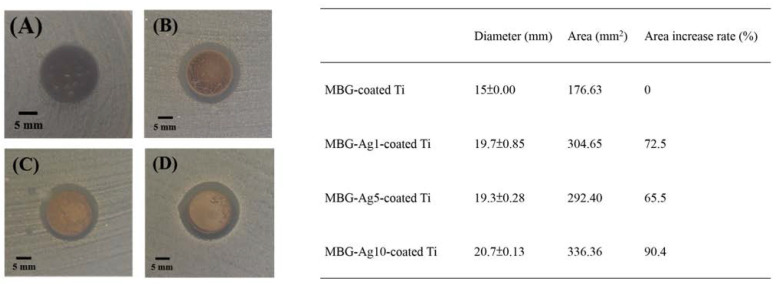
The inhibition zones of (**A**) MBG–coated Ti, (**B**) MBG–Ag1–coated Ti, (**C**) MBG–Ag5––coated Ti, and (**D**) MBG–Ag10–coated Ti against 10^7^ CFU/mL *Streptococcus mutans*.

As shown in Figure 10, the results of the disk diffusion test indicated that the normal growth morphology of *Streptococcus mutans* with bacterial concentration of 10^7^ CFU/mL was white with a small colony. In Figure 10A, the control group MBG-coated Ti shows no inhibition zone around MBG–coated Ti, while *Streptococcus mutans* exhibits normal growth. Measured and calculated from Figure 10B,D, the inhibition zone diameters of MBG–Ag1–coated Ti, MBG–Ag5–coated Ti, and MBG–Ag10–coated Ti were 19.7 ± 0.85, 19.3 ± 0.28, and 20.7 ± 0.13 mm, respectively, while their inhibition zone areas were 304.65, 292.4, and 336.36 mm^2^, respectively. The results showed that MBG-Ag-coated Ti exhibited excellent bacteriostatic ability against *Streptococcus mutans*, and its inhibition zone was clean and sterile. Compared with the MBG–Ag10–coated Ti added with 1 and 5 mol% of Ag, the MBG–Ag10–coated Ti added with 10 mol% of Ag exhibited a more evident inhibition zone, with its area increment rate reaching 90.4%.

Comparing the results of the disk diffusion test with those of the previous film analysis, it was found that MBG–Ag10–coated Ti, having the best antibacterial ability, exhibited the highest silver diffraction peak in the XRD analysis, and the film surface had large nano-silver particles. Subsequently, it could be speculated that the nano-silver particles were more likely to destroy *Streptococcus mutans*.

## 3. Discussion

The contact angle measurement results show the surfaces of MBG–Ag–coated Ti to have good wettability and are hydrophilic. This experimental result is consistent with the literature showing that osteocytes are easily attached to hydrophilic surfaces. Therefore, in the material selection of the implant, a hydrophilic surface should be preferred over a hydrophobic surface to improve the overall osseointegration efficiency [17,18]. There is also evidence that a highly hydrophilic surface affects the amount and variation of adsorbed proteins on bioactive surfaces, thus inhibiting bacterial attachment [19,20]. The silver-containing mesoporous bioactive glass film prepared in this study had a good hydrophilic surface and the appropriate properties for implants in guiding the early phase of cell adhesion, spreading, and titanium colonization.

Based on the adhesion strength experimental results, the adhesion strength of the film decreased with increasing silver content. Therefore, it was speculated that when the aggregation degree of nano silver particles was high, the roughness of the interface between the film and titanium substrate increased, resulting in the adhesion strength between the test tape and film being greater than that between the film and titanium substrate. The current acceptable film adhesion strength in the market is level 5B of the Burger’s test. Thus, the adhesion strength of the MBG–coated Ti and MBG–Ag1–coated Ti prepared in this study met the market demand.

From the FTIR analysis results in this study, the MBG–coated Ti and MBG–Ag–coated Ti have Si-O-Si and P-O-P asymmetric stretching vibration absorption peaks at 1000 cm^−1^ and Ti-O-Si stretching vibration peaks at 800–900 cm^−1^, conforming with the results described in the previous literature [21,22,23]. When the bioactive glass formed a film layer on the titanium substrate, silicon oxide interacted to form a network structure, and the overall structure was not changed by adding silver. This result was similar to those of the previous studies on bioactive glass powders. In addition, the Ti-O-Si bond confirmed that the SiO_2_ in the MBG-coated Ti and TiO_2_ of the titanium substrate were chemically bonded to each other. This result indicated that the metal oxide layer could be used as an intermediate layer for binding the glass ceramic and metal substrate, echoing the chemical bonding theory proposed by Kautz in 1936 [24].

To identify the spherical particles on the surface of the silver-containing mesoporous bioactive glass film, EDS was used to analyze the spherical particle and nanoparticle aggregates by single-point analysis. The results showed that the non-granular aggregates were mainly composed of Si and their atomic weight ratio was 43.33 at%. The spherical particle aggregates were mainly composed of Ag and their atomic weight ratio was 90.43 at%. This indicated the spherical particles on the surface of MBG–Ag–coated Ti to be nano-silver particles. Qualitative and semi-quantitative analyses of all groups were performed by EDS surface analysis. These EDS surface analysis results were compared with the SEM images, and it was seen that the nano-silver particles deposited on the surface were a major factor affecting the elemental composition. When the silver content was increased to more than 5 mol%, the atomic number ratio of silver was higher than that of silicon. It was speculated that the nano-silver particles aggregated into micron-sized particles, covering the silicate substrate and making the silicon undetectable by EDS. From the above results, it was confirmed that with the increase in silver content, many nano-silver particles appeared on the surface of MBG–Ag–coated Ti, and the particle size increased from 0.14 to 0.53 μm. After the qualitative and semi-quantitative analysis by EDS, it was determined that the matrix of the film was silicate, whereas calcium, phosphorus, and silver were dispersed and attached to the network structure of silicon oxide.

After comparing Figure 3(1), Figure 3(2), and the JCPDS database with the Jade 6 software, it was found that each group in Figure 3(2) had more crystal planes (002) and hydroxyapatite (211) at the angles of 2θ = 25.8° and 31.7° (JCPDS no. 73-0293), indicating HAp crystallization on the surface of the film that is not affected by the increase in silver composition. It was concluded from the above experimental results that MBG–Ag–coated Ti has the ability to effectively induce HAp crystallization after immersion in SBF for 24 h, and the HAp morphology is not affected by the amount of silver. Furthermore, previous studies found that after the powder was immersed in SBF for 24 h, the HAp crystals on the surface exhibited a flourishing growth state [22]. Therefore, the thickness of the MBG–Ag–coated Ti film was only 6–18 μm, having potentially the same mineralization ability and biocompatibility as the MBG–Ag powder. Compared with the film thickness prepared by the vapor deposition method, since it uses a target to release the ionic material, it can prepare the film as thin as 600 nm [25]. The disadvantage of the vapor deposition method is that the target material is difficult to develop, and its composition is single. Although the MBG–Ag–coated Ti film in this study prepared by the sol–gel technique is not as thick as nanometers by the vapor deposition method, it still has a great advantage in the preparation of multifunctional dental implant film because of the controllable chemical composition of the film. According to the research results of C. Cherde et al., when the film thickness on the implant increased, its adhesion strength decreased. When the film thickness on the implant was less than 50 μm, the film had strong adhesion to the substrate and produces fewer cracks, while when the film thickness on the implant was greater than 50 μm, obvious delamination and peeling from the substrate were observed [26]. Therefore, the thickness of the MBG–Ag–coated Ti film in this study was controlled to be less than 20 µm, which could avoid cracks and peeling due to too thick film.

Partially oxidized nano-silver particles prepared in an oxygen environment have more significant antibacterial properties than reduced nano-silver particles. At the same time, nano-silver particles easily release silver ions in an aerobic environment, and silver ions themselves also have bacteriostatic ability. Many literatures discuss the antibacterial mechanisms of nano-silver particles, three of which are more widely accepted: (1) Silver ions enter bacterial cells and combine with thiol bonds on proteins, resulting in changes in protein activity and interference with downstream biochemical reactions, or affecting protein maturation. If the important proteins on the electron respiration transport chain on the bacterial cell membrane are destroyed, it will hinder the electron transfer on the respiratory chain, resulting in the change of the hydrogen ion concentration gradient and affecting the efficiency of the ATP synthesis reaction [27]. (2) Ag ions and nano-silver particles induce the generation of reactive oxygen species (ROS) in cells, resulting in oxidative stress and apoptosis. ROS contains single oxygen (O_2_), superoxide (O_2_**^−^**), hydrogen peroxide (H_2_O_2_), and hydroxyl radical (OH·). These substances are inherently present inside and outside cells that perform aerobic respiration, but they are harmful to proteins and DNA. Endogenous reducing agents of bacterial cells, such as NADPH, NADH pools, carotene, ascorbic acid, α-tocopherol, and glutathione (GSH), can deal with these harmful substances. ROS will inhibit the action of glutathione reductase and reduce the amount of glutathione in cells. When the amount of ROS exceeds the load of the endogenous reducing agent glutathione, oxidative stress will form, resulting in protein destruction and deactivation or changes in the balance of important intracellular substances, interfering with normal cell function execution [27,28]. (3) Nano-silver particles directly cause the destruction of bacterial outer membrane, cell wall, and cell membrane. Small holes appear on the bacterial cell wall treated with nano-silver particles, and the appearance of the bacteria is shrinking, deformed, or broken. In addition, nano-silver particles also accumulate on the cell membrane and affect the permeability of materials inside and outside the membrane, resulting in the leakage of materials inside the cell [29]. The inhibition zone results showed MBG–Ag1–coated Ti to have the best antibacterial effect on *Aggregatibacter actinomycetemcomitans*. From the results of XPS analysis, it was observed that MBG–Ag1–coated Ti had a high proportion of silver ions (50.83 at%), while the proportion of silver ions in both MBG–Ag5–coated Ti and MBG–Ag10–coated Ti were relatively low, thereby inferring that MBG-Ag1-coated Ti released more silver ions than MBG–Ag5–coated Ti and MBG–Ag10–coated Ti. Therefore, MBG–Ag1–coated Ti showed a larger diameter and area of inhibition zone in the disk diffusion results. It was also speculated that the infection causing bacteria in the early stage of peri-implantitis (*Aggregatibacter actinomycetemcomitans*) were more susceptible to death due to the influence of silver ions than silver atoms. Its antibacterial mechanism is more in line with the first and second antibacterial mechanisms of nano-silver particles listed above. Comparing the results of the disk diffusion test analysis against *Streptococcus mutans*, it was found that MBG–Ag10–coated Ti, having the best antibacterial ability, exhibited the highest silver diffraction peak in the XRD analysis, and the film surface had large nano-silver particles. Subsequently, it could be speculated that the nano-silver particles were more likely to destroy *Streptococcus mutans*. Its antibacterial mechanism is more in line with the third antibacterial mechanisms of nano-silver particles listed above.

## 4. Materials and Methods

### 4.1. Material Preparation

80SiO_2_–15CaO–5P_2_O_5_ mesoporous bioactive glass precursor solutions containing 1, 5, and 10 mol% of silver were prepared using the sol–gel technique [30]. The synthetic process included the use of alcohol as a solvent, 2 M nitric acid as an acid-base regulator, nonionic surfactant Pluronic F127 (EO_106_-PO_70_-EO_106_) (CRODA, Montigny-Le-Bretonneux, France) as a templated surfactant, tetraethyl orthosilicate (C_8_H_20_O_4_Si, TEOS) (Thermo scientific, Dreieich, Germany) as a structural scaffold and silicon precursor; calcium nitrate tetrahydrate (Ca(NO_3_)2.4H_2_O) (Showa, Tokyo, Japan), triethyl phosphate (C_6_H_15_O_4_P, TEP) (Alfa Aesar, Boston, MA, USA), and silver nitrate (AgNO_3_) (Showa, Tokyo, Japan) were used as the precursors of calcium, phosphorus, and silver, respectively. All the components were stirred at room temperature for 24 h in the dark to form silver-containing mesoporous bioactive glass precursor solutions (MBG–Ag).

### 4.2. Titanium Implant Specimen Preparation

In this study, Grade IV pure titanium discs with SLA surfaces having a diameter and thickness of 15 and 3 mm, respectively, were used as the specimens. The SLA surfaces were prepared by sandblasting the turned surfaces with large-grit alumina particles in the size range of 250–500 µm. Subsequently, the surfaces were chemically treated with sulfuric and hydrochloric acids [31]. Topographically, the SLA surfaces achieved a roughness with large dips, sharp edges, and small micro pits, increasing their contact surface with the subsequent formation of the antibacterial film and exhibiting efficient osseointegration when implanted in vivo [32,33].

The titanium implant specimens were pretreated by immersing them in acetone and cleaning them with an ultrasonic shaker for 10 min to remove the impurities and dust on their surfaces. Next, they were immersed in 75% alcohol, shaken, and sterilized for 10 min. The samples were then immersed in deionized water, shaken, cleaned for 10 min, and finally dried to obtain blank specimens, hereafter referred to as SLA Ti. Before spin coating the MBG–Ag, the pretreated SLA Ti needed to be oxidized at 650 °C for 1 h to obtain specimens of the control group, hereinafter referred to as oxide Ti.

Next, 100 μL of the precursor solution (MBG–Ag) was added dropwise to the center of each SLA Ti specimen, and the parameters of the spin coater (SP102, Olink Technology Co., Ltd., New Taipei City, Taiwan) were set at 500 rpm for 60 s for film coating. The coated specimens were placed in a fume hood to age for 24 h, then moved to a 60 °C oven to dry for 24 h, and finally set at a heating rate and temperature of 10 °C/min and 500 °C, respectively, for the heat treatment to remove the impurities and template, thereby obtaining silver-containing mesoporous bioactive glass films on the coated specimens. These specimens with a silver composition of 1 mol%, 5 mol%, and 10 mol% are hereafter referred to as MBG–Ag1–coated Ti, MBG–Ag5–coated Ti, and MBG–Ag10–-coated Ti. A mesoporous bioactive glass film without silver was used as the control group, hereafter referred to as MBG–coated Ti.

### 4.3. Properties of Silver-Containing Mesoporous Bioactive Glass Film

#### 4.3.1. Wettability Analysis of Silver-Containing Mesoporous Bioactive Glass Film

A contact angle measurement system (DSA100, KRÜSS Scientific, Hamburg, Germany) was used to measure the hydrophilicity and hydrophobicity of the film surfaces. The sessile drop method was employed, which estimates the wettability of a local area on a solid surface and directly measures the contact angle between the droplet baseline and liquid/solid-interface tangent at the liquid/solid/gas-triple contact point. Using distilled water as the test liquid, the specimens were washed with distilled water and then dried for 24 h in an environment with an experimental temperature of 20 ± 5 °C and relative humidity of 50 ± 10%. The contact angles on the surface of the specimens were then measured. Five different surface contact angles were measured and averaged for each sample.

The surface wettability of MBG–Ag1–coated Ti, MBG–Ag5–coated Ti, and MBG–Ag10–coated Ti was measured, and MBG–coated Ti and SLA Ti were used as the control groups.

#### 4.3.2. Adhesion Strength Analysis of Silver-Containing Mesoporous Bioactive Glass Film

ASTM D–3359 “Standard Test Method for Adhesion Measurement by Tape” (Burger test) was used to evaluate the adhesion strength of the silver-containing mesoporous bioactive glass film on the titanium substrate. The Burger’s test was divided into methods A and B according to the thickness of the films. In this study, the thickness of the films was approximately 5–15 µm. Therefore, the B method was used to determine the adhesion strength of the films. Method B used a diamond knife to scribe six lines of the prescribed pattern on the silver-containing mesoporous bioactive glass films and reach the SLA Ti substrate, wherein each line was tested at an interval of 1 mm. A 3M–600 tape was placed on the prescribed pattern using an eraser to compress the tape. After ~60 s, the tape was swiftly pulled away from the surface of the films at an angle of 180°. The test results were obtained using a USB digital microscope (Model:UM05-CAM, Vitiny, Kaohsiung, Taiwan).

### 4.4. Characterization of Silver-Containing Mesoporous Bioactive Glass Film

#### 4.4.1. X-ray Diffraction (XRD) Measurement

The crystallographic properties of the silver-containing mesoporous bioactive glass films were determined by XRD scanning and analysis. The XRD instrument was a Shimadzu XRD 6000 (Tokyo, Japan) with a Cu-Kα radiation (λ = 1.5432 Å), voltage of 30 kV, current of 20 mA, scan range 2θ of 10°–80°, and step size of 4°/min. The data obtained from the scans were analyzed using the Jade 6.5 XRD software (ICDD, Newtown Square, PA, USA), and the crystal structures were confirmed using the Joint Committee on Powder Diffraction Standards Card (JCPDS) database.

#### 4.4.2. Fourier Transform Infrared Spectroscopy (FTIR) Confirmation

From the FTIR absorption spectra, both the structure of molecules and nature of vibrational bonds or rotational bonds were determined, and the presence and content of compounds were also identified and analyzed.

In this study, an FTIR instrument (Nicolet 6700, Thermo Fisher Scientific, Waltham, MA, USA) was used to conduct the experiments, and was based on the total reflection attenuation in the infrared light spectrum with a wavenumber range of 2000–650 cm^−1^ to analyze the functional groups of the silver-containing mesoporous bioactive glass film.

#### 4.4.3. Scanning Electron Microscopy (SEM) and Energy Dispersion Spectroscopy (EDS) Observation

In this study, SEM was used to observe the surface morphology of the MBG–Ag–coated Ti film, and the corresponding elemental composition was analyzed via EDS. A scanning electron microscope (SEM) (JSM-6330TF, JEOL, Tokyo, Japan) was used to record the images using synchronous acquisition. Energy dispersive X-ray spectroscopy (EDS) (x-stream, INCA, Oxford, UK) equipped with SEM was used to identify the constituent elements of the silver-containing mesoporous bioactive glass film.

In the pretreatment step, the titanium implant specimens were dried for 3–5 days to completely evaporate the water in the specimens, and then gold-plated for 300 s. The surface morphology and cross-sectional shape of the specimens were observed, and their corresponding film thickness (µm) was calculated from the SEM images. Three points were measured for each specimen, and a total of 3–4 specimens were used to calculate the average value.

#### 4.4.4. X-ray Photoelectron Spectroscopy (XPS) Measurement

In this study, an XPS instrument (5000 Versaprobe, ULVAC-PHI, Inc., Kanagawa, Japan) was used to detect the ionic and atomic state ratios of Ag on the surface of the silver-containing mesoporous bioactive glass film, and the spectrum was divided using the CasaXPS software to confirm the valence state of Ag as the antibacterial source.

### 4.5. In Vitro Bioactivity Assay of Silver-Containing Mesoporous Bioactive Glass Film

In this study, silver-containing mesoporous bioactive glass films were immersed in an SBF for an in vitro biological activity test. Hench et al. observed that an SiO_2_ and calcium phosphate layer formed on the surface of the bioactive glass by itself, and ascertained that thus, it could be combined with human bone tissues [34]. The titanium implant specimens were immersed in SBF at 37 °C (simulating human body temperature) and soaked for 24 h. After that, they were removed, rinsed with sterile phosphate-buffered saline (PBS), and dried. The apatite crystals formed on the surface of the titanium implant specimens were identified via XRD.

### 4.6. Bacterial Incubation and Antibacterial Activity of Silver-Containing Mesoporous Bioactive Glass Film

#### 4.6.1. Bacterial Incubation

In this study, *Aggregatibacter actinomycetemcomitans* (American Type Culture Collection, ATCC 29523) and *Streptococcus mutans* (American Type Culture Collection, ATCC 25175), purchased from the Bioresource Collection and Research Center (BCRC, Hsinchu, Taiwan), were inoculated into a brain heart infusion (BHI) broth and agar. The growth conditions for *Aggregatibacter actinomycetemcomitans* included incubation at 37 °C ± 2.5 °C under 5% CO_2_ for 18–24 h. The growth conditions of *Streptococcus mutans* included incubation at 37 °C ± 2.5 °C under an aerobic atmosphere for 24–48 h. Following the instructions of ATCC, the inoculation of *Aggregatibacter actinomycetemcomitans* and *Streptococcus mutans* exhibited excellent activity after opening the freeze-dried tube, mixing the broth medium uniformly with glycerin at a ratio of 85:15, and storing in a refrigerator at −80 °C. The strains were active in the BHI broth or agar at 37 °C for 24–48 h against 3–5 generations using the streaking method.

#### 4.6.2. Disk Diffusion Test

In this study, a disk diffusion test was used to determine the antibacterial effect of the silver-containing mesoporous bioactive glass films with different silver contents on *Aggregatibacter actinomycetemcomitans* and *Streptococcus mutans*, and a bioactive glass film without silver (MBG-coated Ti) was used as the control group.

First, MBG–coated Ti, MBG–Ag1–coated Ti, MBG–Ag5–coated Ti, and MBG–Ag10–coated Ti were uniformly smeared on a solid medium with 10^7^ CFU/mL bacteria. The solid medium was then adjusted to the appropriate incubation time and temperature according to the incubation conditions of the bacteria. After incubation, several areas of the specimens were observed. If there was a ring-shaped clean area around the specimens, it was the antibacterial zone, indicating that the specimens possessed antibacterial ability. Any densely grown bacteria around the specimens indicated that the specimens did not exhibit bacteriostatic ability. The experiment was repeated thrice, and the average diameter and area of the inhibition zone were calculated. The control group was used as the benchmark to calculate the increment rate of the inhibition zone area of the silver-containing mesoporous bioactive glass film using the following formula:

[(inhibition zone area of experimental group) − (area of inhibition zone of control group)]/(area of inhibition zone of control group)

### 4.7. Statistical Analysis

Statistical analyses of the experimental results were performed using a one-way ANOVA. If *p* > 0.05, there was no significant difference between the groups. If *p* < 0.05, there was a significant difference between the groups. Therefore, in the figures, the symbol (*) represents the group with *p* < 0.05, while symbol (**) represents the group with *p* < 0.01.

## 5. Conclusions

From the contact angle measurement results, it was found that MBG–Ag–coated Ti could convert the hydrophobic properties of the surface of the filmless titanium implant into a hydrophilic surface. The adhesion strength test results revealed that the adhesion strength decreased with increasing silver content. The XRD patterns confirmed that an increase in the silver content in MBG–Ag–coated Ti increased the diffraction peak intensity of silver. Through FTIR analysis, it was found that the interaction between silicon oxides formed a network structure, and chemical bonds were formed between the film and titanium implant. The SEM and EDS results showed that with the increase in silver content on the surface of the film, the deposition of nano-silver particles showed a trend of denser and larger size. The XPS analysis showed that Ag^+^/Ag^0^ ratio of the film was observed to decrease with increasing silver content. The in vitro bioactivity test showed MBG-Ag-coated Ti to have good mineralization ability and biocompatibility. After the disk diffusion test, it was found that MBG-Ag-coated Ti exhibited significant antibacterial activity against both *Aggregatibacter actinomycetemcomitans* and *Streptococcus mutans*.

These results suggest new strategies for the surface functionalization of titanium implants with silver-containing mesoporous bioactive glass. Such implant functionalization may be promising for the prevention and treatment of early implant failure and peri-implantitis, considering the potential of MBG–Ag for the controlled release of bioactive and antibacterial agents. Next-generation implants made of SLA Ti infiltrated with mesoporous SiO_2_ did not seem to compromise the osseointegration process. Subsequent studies will explore the use of such Ti/SiO_2_ implants in preventing and treating peri-implantitis, considering their potential for the controlled release of antibiofilm compounds.

## Figures and Tables

**Table 1:** XPS data of MBG–Ag–coated Ti.

Specimen	Element/Transition Peak	Binding Energy (eV)	Peak Area (Counts)	Concentration (at%)
MBG–Ag1–coated Ti	Ag _3d3/2_	374.90	3753.42	20.33
	Ag _3d5/2_	368.90	5630.12	30.49
	Ag(0) _3d3/2_	374.75	3631.98	19.67
	Ag(0) _3d5/2_	368.75	544.97	29.51
MBG–Ag5–coated Ti	Ag _3d3/2_	375.21	3430.62	12.92
	Ag _3d5/2_	369.21	5145.94	19.38
	Ag(0) _3d3/2_	374.41	7192.30	27.08
	Ag(0) _3d5/2_	368.41	10,788.45	40.62
MBG–Ag10–coated Ti	Ag _3d3/2_	374.14	2362.13	15.54
	Ag _3d5/2_	368.14	3543.19	23.31
	Ag(0) _3d3/2_	374.90	3717.13	24.46
	Ag(0) _3d5/2_	368.90	5575.70	36.69

## Data Availability

Not applicable.
